# Prerequisites for effective isolated limb perfusion using tumour necrosis factor alpha and melphalan in rats

**DOI:** 10.1038/sj.bjc.6690335

**Published:** 1999-04

**Authors:** J H W de Wilt, E R Manusama, S T van Tiel, M G A van Ijken, T L M ten Hagen, A M M Eggermont

**Affiliations:** Department of Surgical Oncology, University Hospital Rotterdam Dijkzigt – Daniel den Hoed Cancer Centre, Groene Hilledijk 301, 3075 EA Rotterdam, The Netherlands

**Keywords:** TNF, melphalan, isolated limb perfusion, rats

## Abstract

An isolated limb perfusion (ILP) model using soft tissue sarcoma-bearing rats was used to study prerequisites for an effective ILP, such as oxygenation of the perfusate, temperature of the limb, duration of the perfusion and concentration of tumour necrosis factor (TNF). Combination of 50 μg TNF and 40 μg melphalan demonstrated synergistic activity leading to a partial and complete response rate of 71%. In comparison to oxygenated ILP, hypoxia was shown to enhance anti-tumour activity of melphalan alone and TNF alone but not of their combined use. Shorter perfusion times decreased anti-tumour responses. At a temperature of 24–26°C, anti-tumour effects were lost, whereas temperatures of 38–39°C or 42–43°C resulted in higher response rates. However, at 42–43°C, local toxicity impaired limb function dramatically. Synergy between TNF and melphalan was lost at a dose of TNF below 10 μg in 5 ml perfusate. We conclude that the combination of TNF and melphalan has strong synergistic anti-tumour effects in our model, just as in the clinical setting. Hypoxia enhanced activity of melphalan and TNF alone but not the efficacy of their combined use. For an optimal ILP, minimal perfusion time of 30 min and minimal temperature of 38°C was mandatory. Moreover, the dose of TNF could be lowered to 10 μg per 5 ml perfusate, which might allow the use of TNF in less leakage-free or less inert perfusion settings. © 1999 Cancer Research Campaign


*British Journal of Cancer* (1999) **80**(1/2), 161–166
© 1999 Cancer Research Campaign
Article no. bjoc.1998.0335

### Prerequisites for effective isolated limb perfusion using tumour necrosis factor alpha and melphalan in rats

**JHW de Wilt, ER Manusama, ST van Tiel, MGA van Ijken, TLM ten Hagen and AMM Eggermont**

Department of Surgical Oncology, University Hospital Rotterdam Dijkzigt – Daniel den Hoed Cancer Centre, Groene Hilledijk 301, 3075 EA Rotterdam, The Netherlands

**Summary** An isolated limb perfusion (ILP) model using soft tissue sarcoma-bearing rats was used to study prerequisites for an effective ILP, such as oxygenation of the perfusate, temperature of the limb, duration of the perfusion and concentration of tumour necrosis factor (TNF). Combination of 50 μg TNF and 40 μg melphalan demonstrated synergistic activity leading to a partial and complete response rate of 71%. In comparison to oxygenated ILP, hypoxia was shown to enhance anti-tumour activity of melphalan alone and TNF alone but not of their combined use. Shorter perfusion times decreased anti-tumour responses. At a temperature of 24–26°C, anti-tumour effects were lost, whereas temperatures of 38–39°C or 42–43°C resulted in higher response rates. However, at 42–43°C, local toxicity impaired limb function dramatically. Synergy between TNF and melphalan was lost at a dose of TNF below 10 μg in 5 ml perfusate. We conclude that the combination of TNF and melphalan has strong synergistic anti-tumour effects in our model, just as in the clinical setting. Hypoxia enhanced activity of melphalan and TNF alone but not the efficacy of their combined use. For an optimal ILP, minimal perfusion time of 30 min and minimal temperature of 38°C was mandatory. Moreover, the dose of TNF could be lowered to 10 μg per 5 ml perfusate, which might allow the use of TNF in less leakage-free or less inert perfusion settings.

**Keywords**: TNF; melphalan; isolated limb perfusion; rats

Isolated limb perfusion (ILP) is considered the method of choice for the treatment of patients with multiple in-transit melanoma metastases confined to an extremity (Eggermont, 1996). Melphalan has been the standard drug for this regional treatment because of low regional toxicity (Thompson and Gianoutsos, 1992). ILPs with melphalan or other cytostatic drugs has also been used in the treatment of patients with extremity soft tissue sarcomas, although with little success (Kremetz et al, 1977; Klaase et al, 1989). Therefore, Liénard et al (1992) pioneered the application of high-dose tumour necrosis factor (TNF)-α and interferon gamma (IFN-γ) with melphalan, which was reported to result in very high complete response rates in melanoma patients. The impact of using TNF in this setting has been greatest, however, in the treatment of patients with irresectable extremity soft tissue sarcomas, as response rates and limb salvage rates of more than 80% have been reported in large series of patients destined for amputation of the limb (Eggermont et al, 1996*a*, 1996*b*). The selective destruction of tumour vasculature, resulting in haemorrhagic necrosis of the tumour, has been shown in angiographic and histopathological studies (Renard et al, 1994; Eggermont et al, 1996*a*).

Yet many questions regarding mechanisms or conditional requirements by which ILP with TNF and melphalan are mediated, are not solved. Therefore, a tumour model with a highly aggressive non-immunogenic soft tissue sarcoma in BN rats was developed in our laboratory to address these questions (Manusama et al, 1996). Response after ILP with melphalan and TNF in this model correspond well to what is observed in sarcoma patients in terms of synergy between TNF and melphalan, response rate, and histopathological observations (Manusama et al, 1996; Nooijen et al, 1996*a*). This rat model could therefore serve as a credible model to study mechanisms and determine ways to optimize ILP efficacy for the clinical setting. Here we address requirements for an effective ILP setting, such as temperature of the perfusate and limb, duration of the perfusion, oxygenation of the perfusate and concentration of TNF.

*Received 23 June 1998*
*Revised 4 October 1998*
*Accepted 21 October 1998*

Correspondence to: AMM Eggermont

### MATERIAL AND METHODS

### Animals

Male, inbred BN rats, weighing 250–300 g, obtained from Harlan-CPB (Austerlitz, The Netherlands) were used for isolated limb perfusions. Rats were fed a standard laboratory diet ad libitum (Hope Farms Woerden, The Netherlands) and were housed under standard conditions. The experimental protocols adhered to the rules outlined in the Dutch Animal Experimentation Act (1977) and the published 'Guidelines on the Protection of Experimental Animals' by the Council of the E.C. (1986). The protocol was approved by the committee on Animal Research of the Erasmus University Rotterdam, The Netherlands.

### Melphalan

Melphalan (Alkeran, 50 mg per vial, Wellcome, Beckenham, UK) was diluted in 10 ml solvent. Further dilutions were made in 0.9% sodium chloride to give a concentration of 1 μg $\mu l^{-1}$. A volume of 40 μl (= 40 μg) was added to the perfusion circuit.

### TNF

Recombinant human TNF (rHuTNF) was provided by Boehringer (Ingelheim, Germany) having a specific activity of $5.8 \times 10^7$ U $mg^{-1}$ as determined in the murine L-M cell assay (Kramer and Carver, 1986). Endotoxin levels were < 1.25 endotoxin units (EU) per mg protein. TNF concentrations used were 2, 10 and 50 μg in 5 ml perfusate.

### ILP model

The perfusion technique was performed as described previously (Manusama et al, 1996). Briefly, small fragments (3–5 mm) of the rapidly growing and metastasizing BN-175 soft tissue sarcoma were implanted subcutaneously into the right hind limb. Perfusion was performed at a tumour diameter of 13 mm ± 3 mm at least 7 days after implantation. Animals were anaesthetized with Hypnorm (Janssen Pharmaceutica, Tilburg, The Netherlands) and 50 IU of heparin were injected intravenously to prevent coagulation. To keep the rat's hind limb at a constant temperature, a warm water mattress was applied. Temperature was measured with a temperature probe on the skin covering the tumour and was varied between room temperature (24–26°C), 'mild' hyperthermia (38–39°C) and 'true' hyperthermia (42–43°C). The femoral artery and vein were cannulated with silastic tubing (0.012 inch inner diameter (ID), 0.025 inch outer diameter (OD); 0.025 inch ID, 0.047 inch OD respectively; Dow Corning, Michigan, USA). Collaterals were occluded by a groin tourniquet, and isolation time started when the tourniquet was tightened. An oxygenation reservoir and a roller pump were included into the circuit. The perfusion solution was 5 ml Haemaccel (Behring Pharma, Amsterdam, The Netherlands) and the haemoglobin (Hb) content of the perfusate was 0.9 $mmol\text{-}l^{-1}$. Melphalan and TNF were added as boluses to the oxygenation reservoir. A roller pump (Watson Marlow, Falmouth, UK; type 505 U) recirculated the perfusate at a flow rate of 2.4 ml min. A washout with 5 ml oxygenated Haemaccel was performed at the end of the perfusion. Subsequent tumour growth after perfusion was daily recorded by caliper measurement. Tumour volume was calculated as $0.4(A^2B)$, where A is the minimal tumour diameter and B the diameter perpendicular to A.

### Assessment of TNF concentrations in perfusate

During ILP samples for determination of TNF concentrations were collected from the oxygen reservoir at 0.5, 5, 15 and 30 min. Samples were centrifuged and an aliquot of the supernatant was used for analysis. Enzyme-linked immunosorbent assay (ELISA) for rHuTNF was performed as described by Engelberts et al (1991). In short, a 96-well Immuno-Maxisorp plate was coated with murine anti-human TNF monoclonal antibody (mAb) 61E71. A standard titration curve was obtained by making serial dilutions of a known sample of rHuTNF in normal rat serum. Standards and samples were added to the wells and, after washing, the plates were incubated with a polyclonal rabbit antihuman TNF antiserum, followed by addition of an enzyme-labelled antirabbit reagent and enzyme reaction. The detection limit for human TNF is 20 pg $ml^{-1}$.

### Assessment of melphalan concentrations in perfusate

During ILP, samples for determination of melphalan concentrations were collected from the oxygen reservoir at 0.5, 5, 15 and 30 min. Melphalan was measured by gas chromatography–mass spectrometry (GC-MS) (De Boeck et al, 1997). P-[Bis (2-chloroethyl)amino]-phenylacetic acid methyl ester was used as an internal standard. Samples were extracted over trifunctional C18 silica columns. After elution with methanol and evaporation, the compounds were derivatized with trifluoroacetic anhydride and diazomethane in ether. The stable derivates were separated on a methyl phenyl siloxane GC capillary column and measured selectively by single ion monitoring GC-MS in the positive EI mode.

### Assessment of tumour response

The classification of tumour response was: progressive disease (PD) = increase of tumour volume (> 25%) within 5 days; no change (NC) = tumour volume equal to volume during perfusion (in a range of –25% and +25%); partial remission (PR) = decrease of tumour volume (–25 and –90%); complete remission (CR) = tumour volume 0–10% of volume during perfusion or skin necrosis.

### Assessment of limb function

Limb function was a 'clinical' observation in which the rat's ability to walk and stand on the perfused limb was scored 5 days after ILP. On this scale a severe impaired function (grade 0) means that the rat drags its hindlimb without any function; a slightly impaired function (grade 1) means the rat does not use its hindlimb in a usual matter, but stands on it when rising; an intact function of the hindlimb (grade 2) means a normal walking pattern.

### Statistical analysis

Mann–Whitney *U*-test was used to compare tumour volumes in different animal groups and to compare different tumour responses in different groups. Calculations were performed on a personal computer using Graph PadPrism and SPSS for Windows 95.

### RESULTS

### Synergy between TNF and melphalan

In the present study comprising experiments in 167 rats, the efficacy of ILP with TNF and melphalan as reported previously was confirmed (Figure 1) (Manusama et al, 1996). Synergy was demonstrated for the combination of 50 μg TNF and 40 μg melphalan. At 5 days after ILP a significant difference was observed in mean tumour volume as compared to sham perfusions ($P < 0.001$), TNF perfusions alone ($P < 0.001$) and melphalan perfusion alone ($P < 0.001$). No significant difference was found between all other groups.

### Oxygenation/hypoxia

Differences in tumour response in oxygenated versus hypoxic perfusions are summarized in Table 1. Sham oxygenated and hypoxic perfusions resulted in progressive disease in all animals. In oxygenated TNF perfusions, progressive disease occurred in all animals as well; however, significant anti-tumour effect was observed in hypoxic perfusions with TNF in comparison with TNF alone ($P < 0.001$). Hypoxia also significantly increased the

 *© Cancer Research Campaign 1999*

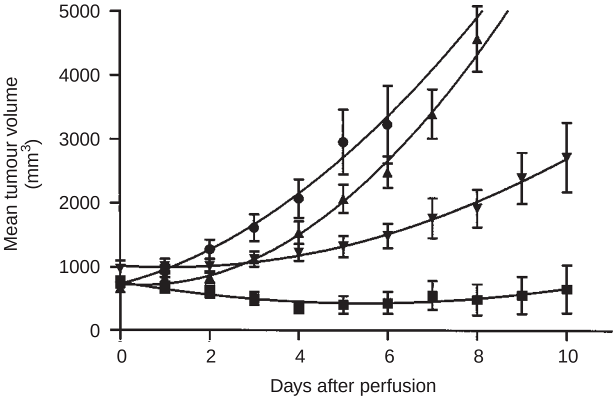

**Figure 1** Growth curves of BN-175 sarcoma after 50 mμg TNF (▲; $n$ = 11), 40 μg melphalan ( ▼ $n$ = 13), TNF plus melphalan (■; $n$ = 28) and sham isolated limb perfusion (•; $n$ = 12). Mean (± s.e.m) of tumour volumes are shown

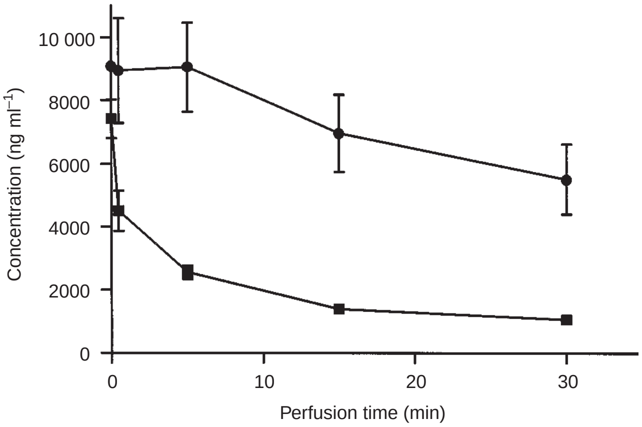

**Figure 2** Concentration (ng ml$^{-1}$) of TNF (•; $n$ = 5) and melphalan (■ ; $n$ = 5) in the perfusate as a function of time in rats. During isolated limb perfusion 50 μg TNF with 40 μg melphalan was added to 5 ml perfusate as a bolus anti-tumour response after ILP with melphalan ($P$ = 0.03) as compared to oxygenated perfusions with melphalan alone. Oxygenated ILP with melphalan and TNF resulted in an overall response rate of 71%. No further improvement of this effect was demonstrated in hypoxic perfusions with the combination of melphalan and TNF. In all hypoxic perfusions, no additional limb toxicity was observed as compared with oxygenated perfusions (data not shown).

### TNF and melphalan concentrations during perfusion

Pharmacokinetic studies of TNF and melphalan in the perfusate were performed, which shows a minimal decrease during perfusion of TNF concentrations, indicating a leakage-free perfusion system and a continuous exposure of the vasculature of high levels of TNF (Figure 2). Melphalan concentrations, on the other hand, decreased dramatically in the first minutes of ILP, indicating rapid uptake by the tissues of the limb (Figure 2).

### Hyperthermia

Standard perfusions with TNF and melphalan were performed with 'mild' hyperthermia (38–39°C). At day 5 after perfusion, rats perfused at room temperature (24–26°C) show almost no anti-tumour effects versus the high response rates seen with 'mild' and 'true' hyperthermic conditions (both $P$ < 0.001). Quality of response (percentage of CR) was not further increased by 'true' hyperthermia in comparison with 'mild' hyperthermia, whereas toxicity to the normal tissues was significantly enhanced leading to increased grade 0 function of the limb (= severe impairment) in this group (Table 2).

### Perfusion time

A gradual, and almost complete, loss of efficacy was observed when perfusion time was reduced from 30 min to 10 min. Table 3 demonstrates the effect of different perfusion times on tumour response of TNF and melphalan. Response rates decreased from 71% to 45% ($P$ < 0.005). Moreover, complete responses decreased from 17 out of 28 animals at 30 min to 0 out of 11 animals at 10 min.

### Minimally required TNF dose

Standard perfusions were performed with 50 μg TNF in 5 ml perfusate on rats that weighed approximately 250 g. A de-escalation study demonstrated similar overall response rates of > 70% at 10 μg (= 40 μg kg$^{-1}$) and 50 μg TNF (= 200 μg kg$^{-1}$) (Table 4). A small (but not significant) drop in complete responses was observed at 10 μg TNF. At 2 μg TNF (= 8 μg kg$^{-1}$) no synergy

**Table 1** Tumour responses of BN-175 sarcoma after isolated limb perfusion with or without hypoxia

| Tumour response | Sham ($n$ = 12) | Sham + hypoxia ($n$ = 10) | TNF ($n$ = 11) | TNF + hypoxia ($n$ = 11) | Melphala Melphalan ($n$ = 13) | Melphalan + hypoxia ($n$ = 10) | Melphalan + TNF ($n$ = 28) | Melphalan + TNF + hypoxia ( $n$ = 18) |
|---|---|---|---|---|---|---|---|---|
| Progressive disease | 12 | 10 | 11 | 3 | 3 | | 2 | 2 |
| No change | | | | 2 | 10 | 7 | 6 | 3 |
| Partial response | | | | 5 | | 3 | 3 | 9 |
| Complete response | | | | 1 | | | 17 | 4 |
| Percentage responses (partial and complete) | | | | 55 | | 30 | 71 | 72 |

Perfusions were performed with 50 μg TNF and 40 μg melphalan under constant temperature (38–39°C) for 30 min.

© Cancer Research Campaign 1999 

**Table 2** Tumour responses of BN-175 sarcoma after isolated limb perfusion with different temperature conditions

| Tumour response | Melphalan + TNF 24–26°C (*n* = 12) | Melphalan + TNF 38–39°C (*n* = 28) | Melphalan + TNF 42–43°C (*n* = 10) |
|---|---|---|---|
| Progressive disease | 6 | 2 | |
| No change | 6 | 6 | |
| Partial response | | 3 | 7 |
| Complete response | | 17 | 3 |
| Percentage responses (partial and complete) | | 71 | 100 |
| Severe impaired limb function (grade 0) | 0/12 (0%) | 6/28 (21%) | 8/10 (80%) |

Perfusions were performed with 50 μg TNF and 40 μg melphalan with oxygenation for 30 min.

**Table 3** Tumour responses of BN-175 sarcoma after isolated limb perfusion with variable perfusion times

| Tumour response | Melphalan + TNF 10 min (*n* = 11) | Melphalan + TNF 20 min (*n* = 10) | Melphalan + TNF 30 min (*n* = 28) |
|---|---|---|---|
| Progressive disease | 3 | 1 | 2 |
| No change | 3 | 3 | 6 |
| Partial response | 5 | 3 | 3 |
| Complete response | | 3 | 17 |
| Percentage responses (partial and complete) | 45 | 60 | 71 |

Perfusions were performed with 50 μg TNF and 40 μg melphalan under constant temperature (38–39°C) and with oxygenation.

**Table 4** Tumour responses of BN-175 sarcoma after isolated limb perfusion with variable TNF concentrations

| Tumour response | 2 μg TNF + 40 μg melphalan (*n* = 10) | 10 μg TNF + 40 μg melphalan (*n* = 10) | 50 μg TNF + 40 μg melphalan (*n* = 28) |
|---|---|---|---|
| Progressive disease | 3 | 1 | 2 |
| No change | 6 | 2 | 6 |
| Partial response | 1 | 3 | 3 |
| Complete response | | 4 | 17 |
| Responses (%) (partial and complete) | 10 | 70 | 71 |

Perfusions were performed under constant temperature (38–39°C), with oxygenation and for 30 min. TNF and melphalan doses are total doses added to 5 ml perfusate as a bolus.

with melphalan was observed, this anti-tumour response differed significantly with 10 μg ($P$ = 0.01) and 50 μg ($P$ < 0.001). No complete remissions, and only one partial response, were observed, which corresponds to what is usually observed after an ILP with melphalan alone. These findings indicate that the dose of TNF can be reduced five- to tenfold without effecting response rates, which might facilitate the use of TNF in less leakage-free settings or at sites more responsive to the toxic effect of TNF.

### DISCUSSION

The non-immunogenic BN-175 soft tissue sarcoma model in BN rats has previously been shown to be an adequate model to address questions for the clinical situation as response patterns to TNF, melphalan and their combination (Manusama et al, 1996). Moreover, histopathologic observations (Nooijen et al, 1996*a*) closely resemble observations in patients (Renard et al, 1994; Nooijen et al, 1996*b*). TNF alone is not active (Posner et al, 1994) and melphalan is only marginally active in soft tissue sarcoma (Kremetz et al, 1977; Klaase et al, 1989), whereas the combination results in very high response rates in this rat model just as in melanoma patients (Liénard et al, 1992; Fraker et al, 1996) or soft tissue sarcoma patients (Eggermont et al, 1996*a*; 1996*b*). Here we report on 167 ILPs to determine ways to optimize ILP efficacy for the clinical setting. Wu et al (1997) previously demonstrated that high flow rate and protein-free perfusate may enhance the effectiveness of ILP with melphalan alone in nude rats. The requirements for a successful ILP such as oxygenation of the perfusate, temperature of the limb, duration of the perfusion and concentration of TNF are addressed in this study. Regarding the factors studied here, results are in line with clinical observations, although these elements have never been studied in a direct comparative fashion in the clinic.

Hypoxia can increase the sensitivity of tumour cells to chemotherapeutic agents since it can cause dividing cells to halt their progression through the cell cycle, by allowing them to progress to and then remain in a G1-like susceptible state (Vaupel et al, 1989). Since Thompson et al (1994) published results of hypoxic ILP, the question whether the much more expensive oxygenated ILPs using a heart–lung machine are really necessary for the treatment of patients with multiple melanoma metastases remains unanswered. We observed that hypoxia enhanced the effects of TNF alone. Similarly, hypoxia enhanced effects of melphalan alone, in line with Skarsgards et al (1995) who reported increased cytotoxicity of melphalan with hypoxia both in vitro and in vivo. Part of the enhancement of the anti-tumour effects by hypoxia could be mediated by the reperfusion injury associated with hypoxic perfusions and absent during oxygenated perfusions. This reperfusion injury apparently would have a preferential effect on the tumour as no impairment of limb function, as a measure of local toxicity, is observed in hypoxic perfusions. Hypoxia did not further increase the efficacy of the combination of TNF and melphalan. Presumably the potential enhancing effect was overshadowed by the synergism of the combination of TNF and melphalan, which again seems to be the central and crucial phenomenon. For the clinical situation it remains an interesting question whether the use of the oxygenator can be abandoned without causing an increase in regional toxicity as has been observed in the hypoxic setting in our model.

The application of hyperthermia in ILP is advocated since it has been shown that the in vivo drug uptake by in-transit metastases is higher at 39.5°C than at 37°C (Omlor et al, 1992) and that hyperthermia enhance anti-tumour effects of melphalan dramatically in vitro (Clark et al, 1994; Robins et al, 1995). Hyperthermia also enhances anti-tumour effects of TNF, as was demonstrated in different tumour models in vitro and in vivo (Niitsu et al, 1988; Watanabe et al, 1988*a*; Klostergaard et al, 1992). We observed that the results in our animal model run parallel to the observations in the clinic. Only perfusions at ‘mild’ or ‘true’ hyperthermia resulted in a synergistic anti-tumour response of TNF and

© Cancer Research Campaign 1999

melphalan, whereas after ILP at 24–26°C all anti-tumour effects were lost. Hyperthermia not only demonstrated to potentiate anti-tumour responses in our animal model, but also increased regional toxicity dramatically when temperatures were above 42°C, which is in line with our clinical experience (Kroon et al, 1992).

There are no clinical or preclinical studies that compare different perfusion times in ILP. Traditionally a perfusion time of 1 h has been adopted for ILPs in patients with melphalan based on pharmacokinetic patterns that request a duration of at least 30 min. The pharmacokinetic profile of melphalan in the perfusate in our model showed a similar rapid decrease in melphalan concentrations as was previously demonstrated by others (Benckhuijsen et al, 1988; Scott et al, 1992). We therefore did not study perfusions longer than 30 min and were more interested if identical results could be obtained after shorter perfusions. It was shown that 30 min is optimal and that lesser efficacy was observed after ILPs of 20 or 10 min. This is probably due to the fact that exposure times over 20 min are needed to get the vascular occlusive and destructive effects of TNF, needed for adequate tumour responses.

TNF is the crucial element in the observed synergy with melphalan. Low-dose TNF has a proliferative effect on angiogenesis, whereas higher doses can cause destruction of newly formed blood vessels (Fajardo et al, 1992). This destruction of blood vessels may lead to thrombocyte aggregation, erythrostasis and haemorragic necrosis found in tumours after treatment with TNF (Watanabe et al, 1988*b*; Renard et al, 1994, 1995; Nooijen et al, 1996*b*). In clinical ILP treatment schedules TNF is used in high doses (4 mg for a lower extremity) to induce the above described effects. This dose is 10- to 50-fold higher than the maximum tolerated dose after intravenous administration in cancer patients (Asher et al, 1987). It was shown that increasing TNF administration did not result in higher response rates but induced regional toxicity to the perfused limb (Fraker et al, 1996). It will obviously add to the safety of the procedure if one can reduce the dose of TNF, while retaining its anti-tumour effects. Pharmacokinetic observations of TNF in the perfusate in this animal model are similar to the clinical situation, in which TNF concentrations remain stable during perfusions (Vrouenraets et al, 1997). Since there is a plateau in TNF levels well above saturation and thus well above threshold level, lower TNF concentrations seems to point to a reasonable action. However, it is not easy to perform a dose de-escalation study in the clinical setting because of the large number of patients needed for such a study. The only publication from Hill et al (1993), in which TNF was used in about five- to sixfold lower concentrations, demonstrated similar high response rates for soft tissue tumours. With a de-escalation study we demonstrated the minimally required TNF dose in our rat model to obtain synergy with melphalan to be 10 μg (= 40 μg $kg^{-1}$).

Since rHuTNF in mice binds only to the p55 receptor and not to the p75 receptor its activity and toxicity is 5–10 times less than murine TNF (MuTNF) (Brouckaert et al, 1992). Also in rats, rHuTNF is at least 5 times more toxic than MuTNF as we established that an intravenous dose of 40 μg MuTNF is lethal in rats (Scheringa et al, 1989), whereas doses of 200 μgr HuTNF are not lethal (own observations). This phenomenon can be explained if we assume that the same receptor binding pattern in rats and mice exists and that activity and toxicity of rHuTNF would be 5–10 times less in rats as well. Therefore, 40 μg $kg^{-1}$ rHuTNF in the rat corresponds roughly with 4–8 μg $kg^{-1}$ rHuTNF in the human setting and thus indicates that the dose of TNF currently used in the clinical setting (approximately 50 μg $kg^{-1}$) may well be reduced five- to tenfold while retaining synergy. Our experiments show that further reduction leads to the complete loss of TNF activity and thus to complete loss of synergy with melphalan. These findings might be of clinical relevance in more than one way. First, it suggests that the very high doses used presently in the clinical setting may well be reduced while retaining efficacy, but even more importantly this observation increases the chances that TNF may become applicable in other settings such as isolated hepatic perfusions (Fraker et al, 1994; Borel Rinkes et al, 1997; Alexander et al, 1998; De Vries et al, 1998). These settings are clearly less ideal, due to local toxicity, than the setting of the ‘inert’ limb perfusion.

In conclusion, our ILP rat model demonstrates strong synergistic anti-tumour effects when TNF is combined with melphalan. We identified as basic requirements for an effective ILP a duration of 30 min and temperature of above 38°C, while hyperthermia above 42°C resulted in unacceptable damage to the normal tissues. Hypoxia was shown to enhance activity of melphalan and TNF alone but did not further improve results of their combined use. The minimally required dose of TNF to induce synergy with melphalan was 10 μg (= 40 μg $kg^{-1}$). These findings may serve as important guidelines for further developments in ILP in the clinical setting.

### ACKNOWLEDGEMENTS

This work was financed in part by a grant from the Dutch Cancer Society. The authors gratefully acknowledge Gert de Boeck for performing melphalan concentration measurements and Ann Seynhaeve for performing TNF–ELISA. Boehringer Ingelheim GmbH is acknowledged for generously providing TNF.

### REFERENCES

Alexander HR, Bartlett DL, Libutti SK, Fraker DL, Moser T and Rosenberg SA (1998) Isolated hepatic perfusion with tumor necrosis factor and melphalan for unresectable cancers confined to the liver. *J Clin Oncol* **12**: 1479–1489

Asher AL, Mule JJ, Reichert CM, Shiloni E and Rosenberg SA (1987) Studies of the antitumor efficacy of systemically administered recombinant tumor necrosis factor against several murine tumors in vivo. *J Immunol* **138**: 963–974

Benckhuijsen C, Kroon BBR, Van Geel AN and Wieberdink J (1988) Regional perfusion treatment with melphalan for melanoma in a limb: an evaluation of drug kinetics. *Eur J Surg Oncol* **14**: 157–163

Borel Rinkes IHM, de Vries MR, Jonker AM, Swaak TJG, Hack CE, Nooijen PTGA, Wiggers T and Eggermont AMM (1997) Isolated hepatic perfusion in the pig with TNF-α with and without melphalan. *Br J Cancer* **75**: 1447–1453

Broukaert P, Libert C, Everaerdt B and Fiers W (1992) Selective species specificity of tumor necrosis factor for toxicity in the mouse. *Lymphokine Cytokine Res* **11**: 193–196

Clark J, Grabs AJ, Parson PG, Smithers BM, Addison RS and Roberts MS (1994) Melphalan uptake, hyperthermic synergism and drug resistance in a human cell culture model for the isolated limb perfusions of melanoma. *Melanoma Res* **4**: 365–370

De Boeck G, van Cauwenberghe K, Eggermont AMM, van Oosterom AT and de Bruijn EA (1997) Determination of melphalan and hydrolysis products in body fluids by GC-MS. *J High Res Chromat* **20**: 697–700

De Vries MR, Borel Rinkes IHM, van de Velde CJH, Wiggers T, Tollenaar RAEM, Kuppen PJK, Vahnmeijer A and Eggermont (1998) Isolated hepatic perfusion with tumor necrosis factor-α (TNFα) and melphalan. *Rec Results Cancer Res* **147**: 107–119

Eggermont AMM (1996) Treatment of melanoma in-transit metastases confined to the limb. *Cancer Surv* **26**: 335–349

Eggermont AMM, Schraffordt Koops H, Liénard D, Kroon BBR, van Geel AN, Hoekstra HJ and Lejeune FJ (1996*a*) Isolated limb perfusion with high-dose tumor necrosis factor-α in combination with interferon-γ and melphalan for non-resectable extremity soft tissue sarcomas: a multicenter trial. *J Clin Oncol* **14**: 2653–2665

Eggermont AMM, Schraffordt Koops H, Klausner J, Kroon BBR, Schlag PM, Liénard D, van Geel AN, Hoekstra HJ, Meller I, Nieweg OE, Kettelhack C, Ben-Ari G, Pector JC and Lejeune FJ (1996*b*) Isolated limb perfusion with tumor necrosis factor and melphalan for limb salvage in 186 patients with locally advanced soft tissue extremity sarcomas: the cumulative multicenter European experience. *Ann Surg* **224**: 756–765

Engelberts I, Moller A, Schoen GJ, van der Linden CJ and Buurman WA (1991) Evaluation of measurement of human TNF in plasma by ELISA. *Lymphokine Cytokine Res* **10**: 69–76

Fajardo LF, Kwan HH, Kowalski J, Prionas SD and Allison AC (1992) Dual role of tumor necrosis factor-α in angiogenesis. *Am J Pathol* **140**: 539–544

Fraker DL, Alexander HR and Thom AK (1994) Use of tumor necrosis factor in isolated hepatic perfusion. *Circ Shock* **44**: 45–50

Fraker DL, Alexander HR, Andrich M and Rosenburg SA (1996) Treatment of patients with melanoma of the extremity using hyperthermic isolated limb perfusion with melphalan, tumor necrosis factor, and interferon gamma: results of a tumor necrosis factor dose-escalation study. *J Clin Oncol* **14**: 479–489

Hill S, Fawcett WJ, Sheldon J, Soni N, Williams T and Thomas JM (1993) Low-dose tumour necrosis factor α and melphalan in hyperthermic isolated limb perfusion. *Br J Surg* **80**: 995–997

Klaase JM, Kroon BBR, Benckhuijsen C, van Geel AN, Albus-Lutter CE and Wieberdink J (1989). Results of regional isolation perfusion with cytostatics in patients with soft tissue tumors of the extremities. *Cancer* **64**: 616–621

Klostergaard J, Leroux E, Siddik ZH, Khodadadian M and Tomasovic SP (1992) Enhanced sensitivity of human colon tumor cell lines in vitro in response to thermochemoimmunotherapy. *Cancer Res* **52**: 5271–5277

Kramer SM and Carver ME (1986) Serum-free in vitro bioassay for the detection of tumor necrosis factor. *J Immunol Methods* **93**: 201–206

Krementz ET, Carter RD, Sutherland CM and Hutton I (1977) Chemotherapy of sarcomas of the limbs by regional perfusion. *Ann Surg* **185**: 555–564

Kroon BBR, Klaase JM, van Geel AN and Eggermont AMM (1992) Application of hyperthermia in regional isolated perfusion for melanoma of the limbs. *Reg Cancer Treat* **4**: 223–226

Liénard D, Ewalenko P, Delmotte JJ, Renard N and Lejeune FJ (1992) High-dose recombinant tumor necrosis factor alpha in combination with interferon gamma and melphalan in isolation perfusion of the limbs for melanoma and sarcoma. *J Clin Oncol* **10**: 52–60

Manusama ER, Nooijen PTGA, Stavast J, Durante NMC, Marquet RL and Eggermont AMM (1996) Synergistic antitumour effect of recombinant human tumour necrosis factor α with melphalan in isolated limb perfusion in the rat. *Br J Surg* **83**: 551–555

Niitsu Y, Watanabe, Umeno H, Sone H, Neda H, Yamauchi N, Maeda M and Urushizaki I (1988) Synergistic effects of recombinant human tumor necrosis factor and hyperthermia on in vitro cytotoxicity and artificial metastasis. *Cancer Res* **48**: 654–657

Nooijen PTGA, Manusama ER, Eggermont AMM, Schalkwijk L, Stavast J, Marquet RL, de Waal RMW and Ruiter DJ (1996*a*) Synergistic anti-tumour effects of TNF-α and melphalan in an isolated limb perfusion model of rat sarcoma: a histopathologic, immunohistochemical and electron microscopic study. *Br J Cancer* **74**: 1908–1915

Nooijen PTGA, Eggermont AMM, Verbeek MM, Schalkwijk L, Buurman WA, de Waal RMW and Ruiter DJ (1996*b*) Transient induction of E-selectin expression following TNFalpha-based isolated limb perfusion in melanoma and sarcoma patients is not tumor specific. *J Immunother Emphasis Tumor Immunol* **19**: 33–44

Omlor G, Gross G, Ecker KW, Burger I and Feifel G (1992) Optimization of isolated hyperthermic limb perfusion. *World J Surg* **16**: 1117–1119

Posner M, Liénard D, Lejeune FJ, Rosenfelder D and Kirkwood J (1994) Hyperthermic isolated limb perfusion (HILP) with tumor necrosis factor alpha (TNF) alone for metastatic in-transit melanoma. *Proc Annu Meet Am Soc Clin Oncol* **13**: A1351

Renard N, Liénard D, Lespagnard L, Eggermont A, Heimann R and Lejeune F (1994) Early endothelium activation and polymorphonuclear cell invasion precede specific necrosis of human melanoma and sarcoma treated by intravascular high-dose tumour necrosis factor alpha (rTNFα). *Int J Cancer* **57**: 656–663

Renard N, Nooijen PTGA, Schalkwijk L, de Waal RMW, Eggermont AMM, Liénard D, Kroon BBR, Lejeune FJ and Ruiter DJ (1995) VWF release and platelet aggregation in human melanoma after perfusion with TNFα. *J Pathol* **176**: 279–287

Robins HI, d'Oleire F, Kutz M, Bird A, Schmitt-Tiggelaar CL, Cohen JD and Spriggs DR (1995) Cytotoxic interactions of tumor necrosis factor, melphalan and 41.8°C hyperthermia. *Cancer Lett* **89**: 55–62

Scheringa M, Keizer A, Jeekel J and Marquet RL (1989) Anti-tumor effect of recombinant TNF-alpha (rMuTNFα) given by continuous i.v. infusion as compared to repeated i.v. injections in a rat metastasis model. *Int J Cancer* **43**: 905–909

Scott RN, Kerr DJ, Blackie R, Hughes J, Burnside G, Mackie RM, Byrne DS and McKay AJ (1992) The pharmacokinetic advantages of isolated limb perfusions with melphalan for malignant melanoma. *Br J Cancer* **66**: 159–166

Skarsgard LD, Skwarchuk MW, Vinczan A, Kristl J and Chaplin DJ (1995) The cytotoxicity of melphalan and its relationship to pH, hypoxia and drug uptake. *Anticancer Res* **15**: 219–224

Thompson JF and Gianoutsos MP (1992) Isolated limb perfusion for melanoma: effectiveness and toxicity of cisplatin compared with that of melphalan and other drugs. *World J Surg* **16**: 227–233

Thompson JF, Waugh RC, Saw RPM and Kam PCA (1994) Isolated limb infusion with melphalan for recurrent limb melanoma: a simple alternative to isolated limb perfusion. *Reg Cancer Treat* **7**: 188–192

Vaupel P, Kallinowski F and Okunieff P (1989) Blood flow, oxygen and nutrient supply, and metabolic micro environment of human tumors: a review. *Cancer Res* **49**: 6449–6465

Vrouenraets BC, Kroon BBR, Ogilvie AC, van Geel AN, Nieweg OE, Swaak AJG and Eggermont AMM (1997) Absence of severe systemic toxicity after leakage controlled isolated limb perfusion (ILP) with high-dose TNFα and melphalan. *Melanoma Res* **7**: s111

Watanabe N, Niitsu Y, Umeno H, Sone H, Neda H, Yamauchi N, Maeda M and Urushizaki I (1988*a*) Synergistic cytotoxic and antitumor effects of recombinant human tumor necrosis factor and hyperthermia. *Cancer Res* **48**: 650–653

Watanabe N, Niitsu Y, Umeno H, Kuriyama H, Neda H, Yamauchi N, Maeda M and Urushizaki (1988*b*) Toxic effect of tumor necrosis factor on tumor vasculature in mice. *Cancer Res* **48**: 2179–2183

Wu ZY, Smithers BM, Parsons PG and Roberts MS (1997) The effects of perfusion conditions on melphalan distribution in the isolated perfused rat hindlimb bearing a human melanoma xenograft. *Br J Cancer* **75**: 1160–1166

*© Cancer Research Campaign 1999*
